# Smart Delivery of Biomolecules Interfering with Peri-Implant Repair in Osteoporotic Rats

**DOI:** 10.3390/ijms25168963

**Published:** 2024-08-17

**Authors:** Laura Vidoto Paludetto, Naara Gabriela Monteiro, Isadora Breseghello, Fábio Roberto de Souza Batista, Cristina Antoniali, Paulo Noronha Lisboa-Filho, Roberta Okamoto

**Affiliations:** 1 Department of Basic Sciences, Araçatuba Dental School, São Paulo State University Júlio de Mesquita Filho—UNESP, Aracatuba 16015-050, SP, Brazil; laura.paludetto@unesp.br (L.V.P.); naara.monteiro@unesp.br (N.G.M.); isadora.breseghello@unesp.br (I.B.); fabio.rs.batista@unesp.br (F.R.d.S.B.); cristina.antoniali@unesp.br (C.A.); 2 Department of Physics and Meteorology, Bauru Sciences School, São Paulo State University Júlio de Mesquita Filho—UNESP, Bauru 17033-360, SP, Brazil; paulo.lisboa@unesp.br

**Keywords:** osteoporosis, dental implants, zoledronic acid, teriparatide, ruterpy

## Abstract

Bisphosphonates are widely used for the treatment of postmenopausal osteoporosis; however, they cause several long-term side effects, necessitating the investigation of local ways to improve osseointegration in compromised bone tissue. The purpose of this study was to evaluate peri-implant bone repair using implants functionalized with zoledronic acid alone (OVX ZOL group, n = 11), zoledronic acid + teriparatide (OVX ZOL + TERI group, n = 11), and zoledronic acid + ruterpy (OVX ZOL + TERPY group, n = 11) compared to the control group (OVX CONV, n = 11). Analyses included computer-assisted microtomography, qualitative histologic analysis, and real-time PCR analysis. Histologically, all functionalized surfaces improved peri-implant repair, with the OVX ZOL + TERI group standing out. Similar results were found in computerized microtomography analysis. In real-time PCR analysis, however, the OVX ZOL and OVX ZOL + TERPY groups showed better results for bone formation, with the OVX ZOL + TERPY group standing out, while there were no statistical differences between the OVX CONV and OVX ZOL + TERI groups for the genes studied at 28 postoperative days. Nevertheless, all functionalized groups showed a reduced rate of bone resorption. In short, all surface functionalization groups outperformed the control group, with overall better results for the OVX ZOL + TERI group.

## 1. Introduction

Due to the continuous technological development in the field of medicine and health, and the expansion of access to basic healthcare, especially in developed and developing countries, there is a notable trend towards an increase in the life expectancy of the population [1]. However, increased longevity also has some consequences, such as the increased prevalence of metabolic diseases. Osteoporosis, a metabolic bone disease characterized by increased susceptibility to bone fractures [2], is a condition closely associated with the aging process. With the onset of menopause and andropause, there is a significant decrease in the production of sex hormones, including estrogen, an important inhibitor of bone resorption [3,4].

The increased longevity of the population also leads to increased demand for rehabilitative treatments, including rehabilitation with osseointegratable implants. With a very high success rate [5], dental implants are currently considered the “gold-standard” alternative for the rehabilitation of partially or fully edentulous patients [6,7]. However, the success of the osseointegration process [8] is highly dependent on the organic conditions of the individual and, consequently, their bone tissue. Osteoporosis, in this scenario, will affect this process. According to some authors, the bone from osteoporotic patients will resemble type IV bone (a large amount of poor-quality medullary bone surrounded by a thin layer of cortical bone), and for this reason, the placement of osseointegrable implants in this group of patients will be contraindicated [9]. Although some systematic reviews have addressed the issue of osseointegration in the presence of osteoporosis, concluding that there is no contraindication to this type of treatment in these patients and also that there is no statistical difference in the survival rates of these implants compared to the group without osteoporosis [10,11], there is still no consensus in the literature on this issue. Some studies have shown long-term impairment in implant stability, with greater marginal bone loss around them [12], higher failure rates [11], and in an animal study, lower expression of ECM (extracellular matrix) in the osteoporotic group, which, according to the authors, could compromise biomechanical stability in the long term [9].

The treatment currently considered the “gold standard” for osteoporosis involves the use of systemic bisphosphonates, antiresorptive agents that delay bone turnover [13]. Although they have very desirable effects on bone tissue, such drugs can cause various undesirable side effects in different body systems [14]. Against this background, various medical organizations and associations, such as the International Osteoporosis Foundation (IOF), are discussing the real cost–benefit ratio of the use of such drugs.

Considering a possible future scenario in which there are osteoporotic patients in need of osseointegrable implants for oral rehabilitation and non-users of systemic bisphosphonates, it is imperative to investigate other alternatives to promote the success of osseointegrable implants, such as local alternatives to improve bone tissue, through preclinical studies in animal models.

In this context, the functionalization of the surface of osseointegrable implants with bioactive molecules in order to promote drug delivery from the coatings via the dip-coating technique to improve peri-implant bone repair against systemic perturbations seems to be an excellent line of research, already carried out by our group. This methodology aims to induce local cellular responses that favor peri-implant repair. Among the options of bioactive molecules already tested in animal models by our laboratory, there are teriparatide (PTH 1-34) and ruterpy. Teriparatide (PTH 1-34), a parathyroid hormone analog, is a bone-anabolic agent that, when used intermittently, acts by stimulating osteoblasts to promote greater bone formation. It has been approved for use by the Food and Drug Administration (FDA) in the United States and is commercially available under the name Forteo^®^, usually administered systemically in combination with bisphosphonates for the treatment of osteoporosis [15]. Some previous work from our research group already used teriparatide on the surface of implants, with interesting results regarding peri-implant repair.

Ruterpy ([Ru(terpy)(bdq)NO]^3+^), a substance synthesized by the FCFRP (Faculty of Pharmaceutical Sciences of Ribeirão Preto—University of São Paulo, USP), is a ruthenium complex that acts as a nitric oxide donor. Nitric oxide is critical for bone repair due to its role in vasodilation and angiogenesis. It directly affects the production of vascular endothelial growth factor (VEGF) and inhibits endothelial cell apoptosis. As a nitric oxide donor, ruterpy increases the production of this mediator, thereby promoting bone repair. Like teriparatide, this compound has also been studied by our group and shown to be beneficial for osseointegration. The results of studies with teriparatide and ruterpy have even led to patents (BR 10 2021 019548 7 and BR 10 2021 025891 8).

On the other hand, zoledronic acid, an antiresorptive belonging to the class of nitrogen-containing bisphosphonates (high potency) acting only on bone tissue, is one of the most effective agents for the control of osteoporosis [16]. Although not yet studied by our research group, its high capacity to inhibit bone resorption raises questions about its possible efficacy when used around implants by the dip-coating technique. Moreover, several studies in the literature have used it alone around implants in models of healthy rats, rats with osteoporosis, and rats with estrogen deficiency, with interesting results [17,18,19,20,21,22,23].

The idea of using such bioactive molecules locally around implants by the dip-coating technique, to study their action in peri-implant bone repair against osteoporosis, seems to be of great value, since their action when used systemically is beneficial for such conditions. However, no study available in the literature has used more than one biomolecule around implants with the dip-coating technique. For this reason, the proposal of this study was to evaluate the above-mentioned substances (teriparatide, ruterpy, and zoledronic acid), which act together and synergistically on peri-implant bone through their aggregation on the surface of implants using the dip-coating technique. These implants can be called “intelligent biomolecule release systems” because the release of the aggregated substances will be intelligent. First, an anti-resorptive drug is administered in the bone tissue, inhibiting its resorption, and then an osteo-forming drug or nitric oxide releaser is made available, acting on bone formation or vasodilation. It is worth noting that both responses are of great value in favoring the cellular steps that occur during the bone repair process. Thus, it will be possible to observe whether these new implant surfaces can promote increased or improved actions compared to those already obtained with conventional surfaces, since the associated biomolecules incorporated may have synergistic effects on peri-implant repair due to their ordered release in this bone bed.

The aim of the present study was to evaluate and characterize the peri-implant repair process following local administration of zoledronic acid alone, zoledronic acid + teriparatide, and zoledronic acid + ruterpy by functionalizing implant surfaces using the dip-coating technique in osteoporotic rat models. The aim was to determine whether the local association and ordered release of such biomolecules would be beneficial and have a synergistic action for osseointegration.

## 2. Results

### 2.1. Confirmation of the Efficacy of Bilateral Ovariectomy Surgery

Eight days after bilateral ovariectomy surgery, the estrous cycle of the animals began, confirming the constant diestrus and thus the success of the surgical procedure. Implant placement in the tibial metaphysis was performed 90 days after ovariectomy, by which time osteoporosis was already established [24].

### 2.2. Three-Dimensional Microtomographic (Micro-CT) Results

**Figure 1:**
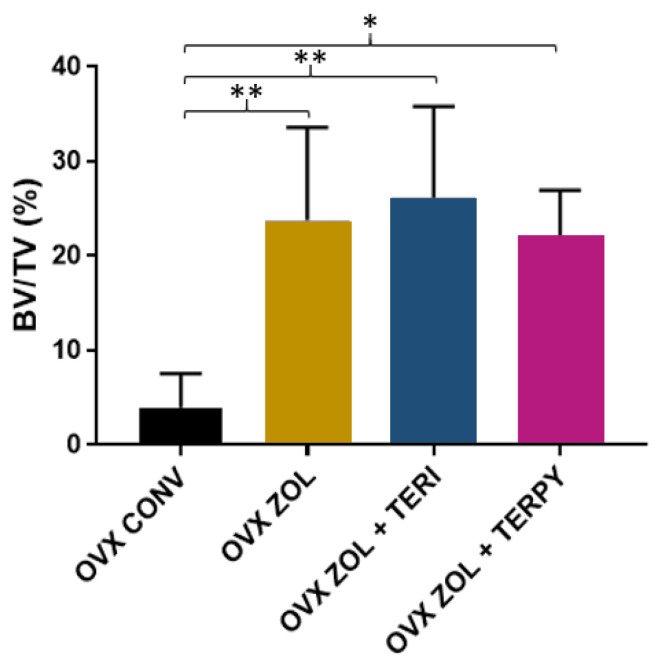
For the BV/TV parameter, there was a statistically significant difference between the OVX CONV group and all other experimental groups (OVX ZOL, OVX ZOL + TERI, and OVX ZOL + TERPY). *: statistically significant difference (*p* ≤ 0.05); **: statistically significant difference (*p* ≤ 0.005).

The mean results obtained for bone volume percentage (BV/TV) were as follows: OVX CONV (3.91% ± 3.61), OVX ZOL (23.77% ± 9.81), OVX ZOL + TERI (26.15% ± 9.62), and OVX ZOL + TERPY (22.09% ± 4.81), as shown in Figure 1.

**Figure 2:**
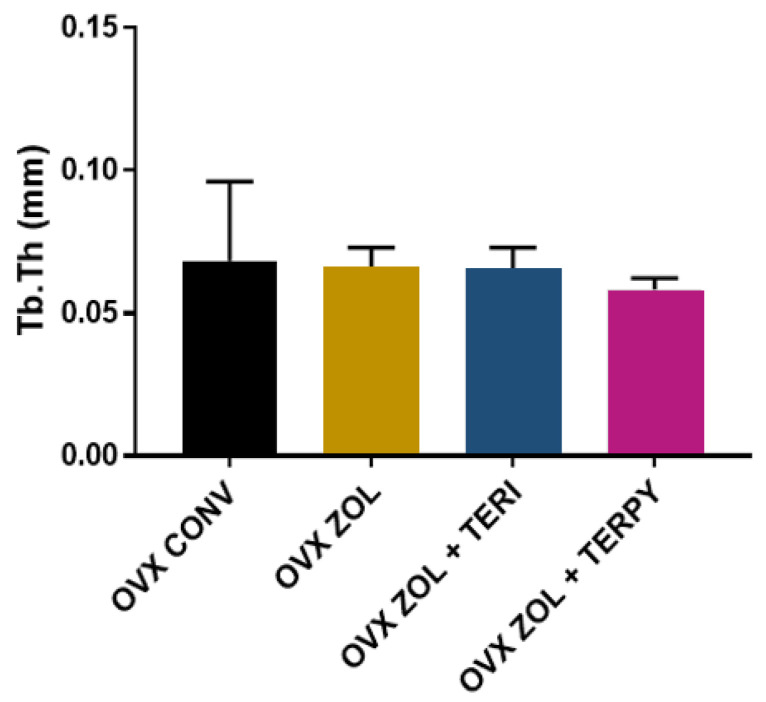
There was no statistically significant difference between the groups for the Tb.Th parameter.

The mean results obtained for trabecular thickness (Tb.Th) were as follows: OVX CONV (0.068 ± 0.027 mm), OVX ZOL (0.066 ± 0.006 mm), OVX ZOL + TERI (0.065 ± 0.007 mm), and OVX ZOL + TERPY (0.058 ± 0.003 mm), as shown in Figure 2.

**Figure 3:**
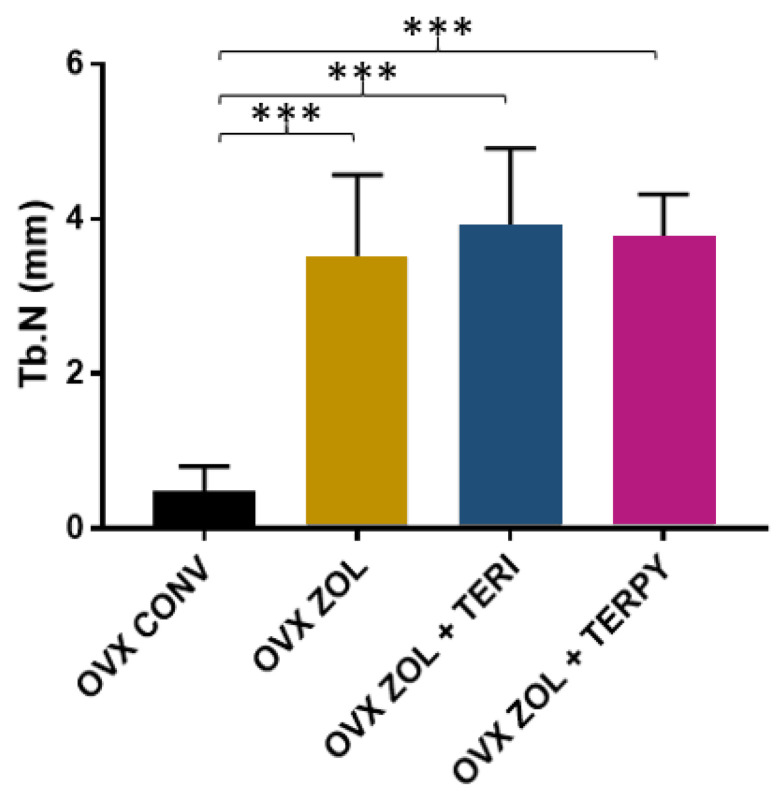
There was a statistically significant difference between the OVX CONV group and all the other experimental groups (OVX ZOL, OVX ZOL + TERI, and OVX ZOL + TERPY) for the Tb.N parameter. ***: statistically significant difference (*p* ≤ 0.0005).

The mean trabecular number (Tb.N) results were as follows: OVX CONV (0.47 ± 0.32 mm), OVX ZOL (3.51 ± 1.05 mm), OVX ZOL + TERI (3.91 ± 1 mm), and OVX ZOL + TERPY (3.76 ± 0.55 mm), as shown in Figure 3.

**Figure 4:**
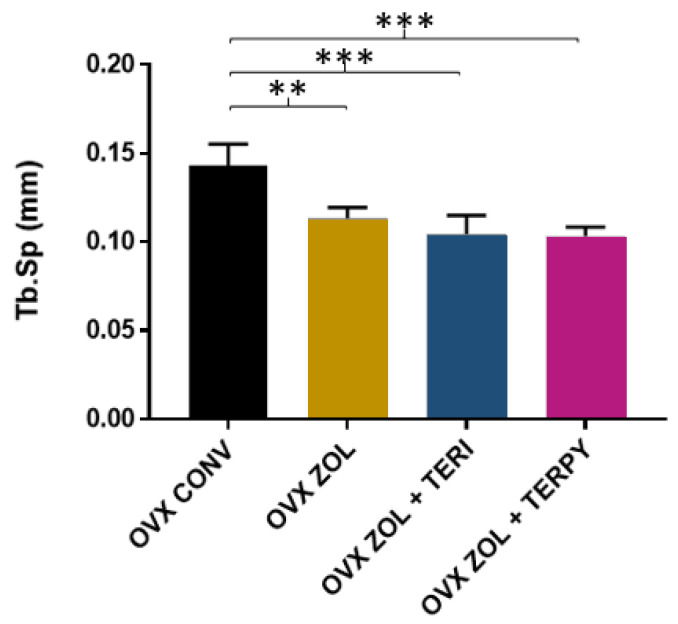
There was a statistically significant difference between the OVX CONV group and all the other experimental groups (OVX ZOL, OVX ZOL + TERI, and OVX ZOL + TERPY) for the Tb.Sp parameter. **: statistically significant difference (*p* ≤ 0.005); ***: statistically significant difference (*p* ≤ 0.0005).

The mean trabecular separation (Tb.Sp) results were as follows: OVX CONV (0.143 ± 0.012 mm), OVX ZOL (0.113 ± 0.006 mm), OVX ZOL + TERI (0.104 ± 0.010 mm), and OVX ZOL + TERPY (0.103 ± 0.005 mm), as shown in Figure 4.

**Figure 5:**
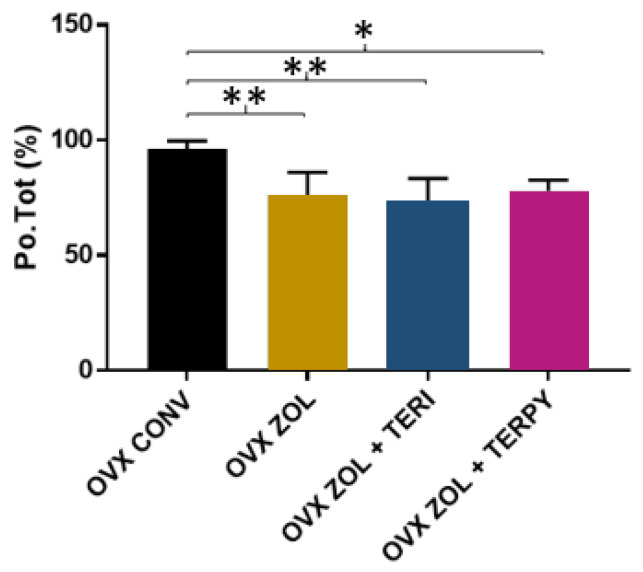
There was a statistically significant difference between the OVX CONV group and all the other experimental groups (OVX ZOL, OVX ZOL + TERI, and OVX ZOL + TERPY) for the Po.tot parameter. *: statistically significant difference (*p* ≤ 0.05); **: statistically significant difference (*p* ≤ 0.005).

The mean results obtained for total bone tissue porosity (Po.Tot) were as follows: OVX CONV (96.09 ± 3.61%), OVX ZOL (76.23 ± 9.81%), OVX ZOL + TERI (73.85 ± 9.62%), and OVX ZOL + TERPY (77.91 ± 4.81%), as shown in Figure 5.

**Figure 6:**
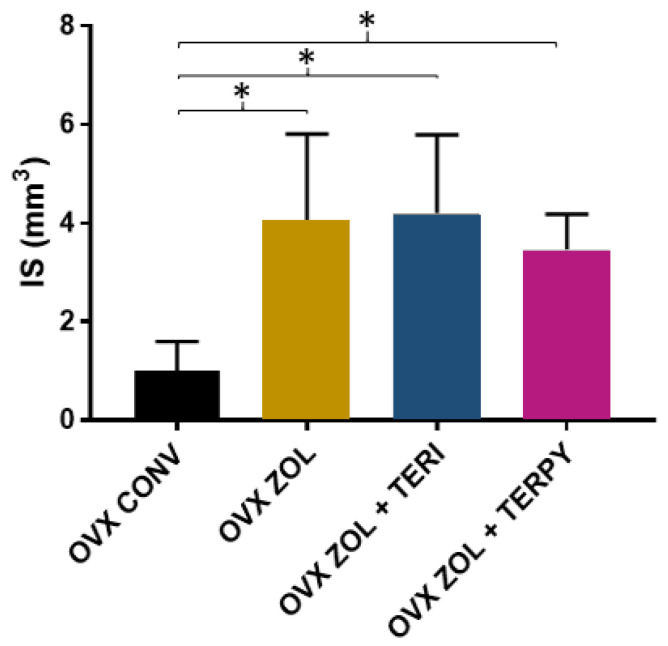
There was a statistically significant difference between the OVX CONV group and all the other experimental groups (OVX ZOL, OVX ZOL + TERI, and OVX ZOL + TERPY) for the I.S parameter. *: statistically significant difference (*p* ≤ 0.05).

The mean results obtained for the bone-to-implant contact surface (I.S) were as follows: OVX CONV (0.99 ± 0.59 mm^3^), OVX ZOL (4.04 ± 1.76 mm^3^), OVX ZOL + TERI (4.21 ± 1.58 mm^3^), and OVX ZOL + TERPY (3.46 ± 0.71 mm^3^), as shown in Figure 6.

All the statistical analysis results for Micro-CT analysis can be found in the Supplementary Materials (Table S1).

### 2.3. Three-Dimensional Microtomographic Reconstructions (CTvox^®^ Software)

**Figure 7:**
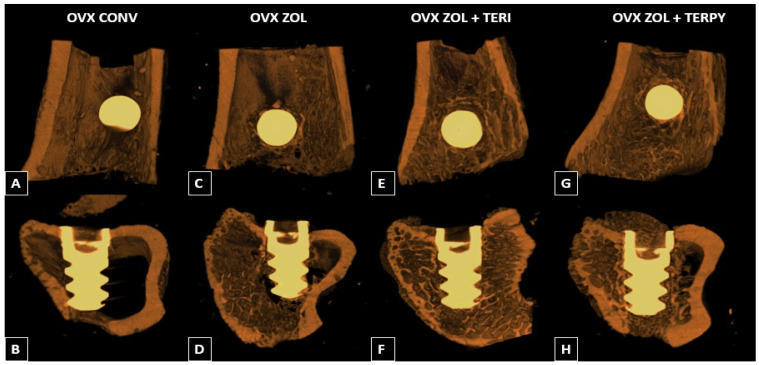
Three-dimensional microtomographic reconstructions of groups OVX CONV (**A**,**B**), OVX ZOL (**C**,**D**), OVX ZOL + TERI (**E**,**F**), and OVX ZOL + TERPY (**G**,**H**) illustrating peri-implant bone formation using the CTvox^®^ software (SkyScan, version 2.7).

Three-dimensional radiographic reconstructions were performed using the CTvox^®^ software (SkyScan, Version 2.7) to illustrate the peri-implant bone formation in the different experimental groups in a didactic manner. We observed greater peri-implant bone formation in the OVX ZOL group compared to the OVX CONV group, and even greater in the OVX ZOL + TERI and OVX ZOL + TERPY groups compared to the OVX CONV and OVX ZOL groups, as shown in Figure 7.

### 2.4. Qualitative Histological Results

#### 2.4.1. OVX CONV Group

**Figure 8:**
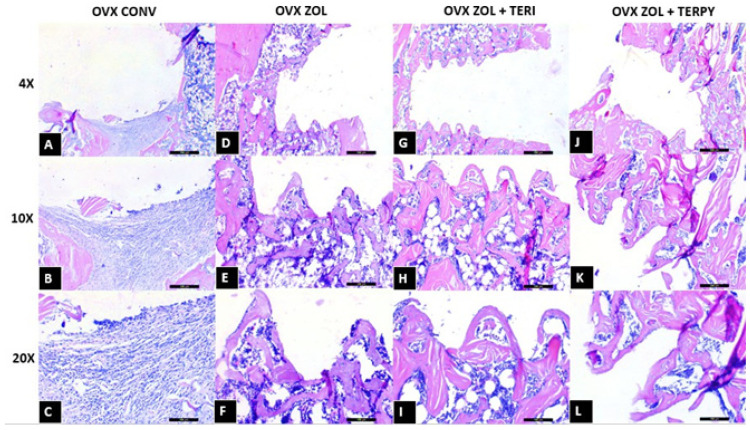
Hematoxylin and eosin staining of OVX CONV (**A**–**C**), OVX ZOL (**D**–**F**), OVX ZOL + TERI (**G**–**I**), and OVX ZOL + TERPY (**J**–**L**) slides at 4×, 10×, and 20× magnifications.

At 28 postoperative days, it was observed that the OVX CONV group had no bone tissue formed around the implant threads. A large amount of disorganized connective tissue was observed at the periphery of the peri-implant region, and only discrete islands of mineralized bone matrix were observed in the cortical region (Figure 8A–C).

#### 2.4.2. OVX ZOL Group

At 28 postoperative days, it was noted that the OVX ZOL group showed the formation of mature bone tissue interspersed with connective tissue around the implant threads, with no predominance between them. It was also possible to observe the morphological definition of the threads (Figure 8D–F).

#### 2.4.3. OVX ZOL + TERI Group

At 28 postoperative days, the OVX ZOL + TERI group showed the predominance of mature bone tissue interspersed with smaller amounts of connective tissue around the implant threads. The morphologic definition of the threads was also clearly visible (Figure 8G–I).

#### 2.4.4. OVX ZOL + TERPY Group

At 28 postoperative days, it was noted that the OVX ZOL + TERPY group also achieved the predominance of mature bone tissue interspersed with smaller amounts of connective tissue around the implant threads. A certain definition of the threads was also observed (Figure 8J–L).

### 2.5. Molecular (Real-Time PCR) Results

**Figure 9:**
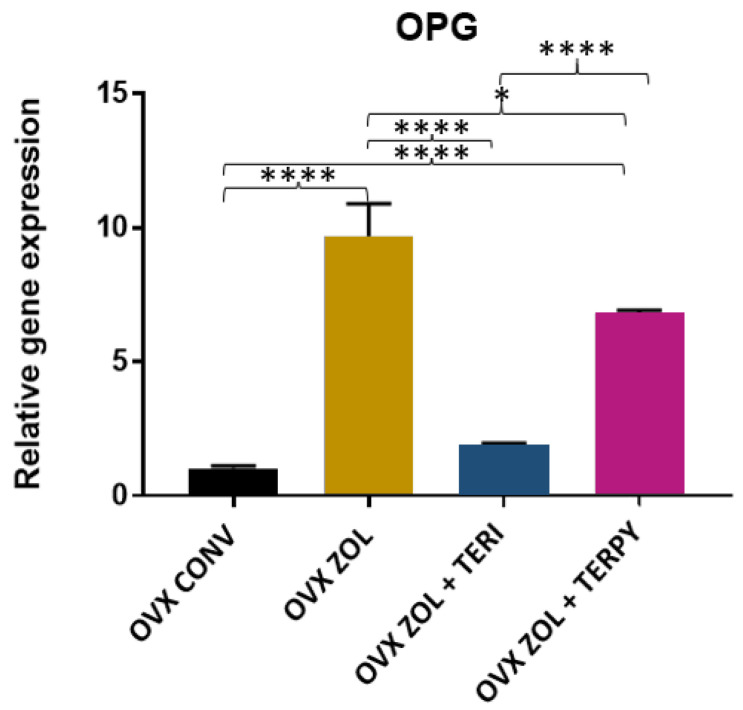
Relative gene expression of the OPG gene for the OVX CONV, OVX ZOL, OVX ZOL + TERI, and OVX ZOL + TERPY groups. Mean values of OVX CONV (1.033), OVX ZOL (9.680), OVX ZOL + TERI (1.917), and OVX ZOL + TERPY (6.813). *: statistically significant difference (*p* ≤ 0.05) and ****: statistically significant difference (*p* ≤ 0.00005).

There was a statistically significant difference in the gene expression of OPG between the following groups: OVX CONV-OVX ZOL, OVX CONV-OVX ZOL + TERPY, OVX ZOL-OVX ZOL + TERI, OVX ZOL-OVX ZOL + TERPY, and OVX ZOL + TERI-OVX ZOL + TERPY, with *p* < 0.005. However, there was no statistically significant difference with the OVX CONV-OVX ZOL + TERI group, as shown in Figure 9.

**Figure 10:**
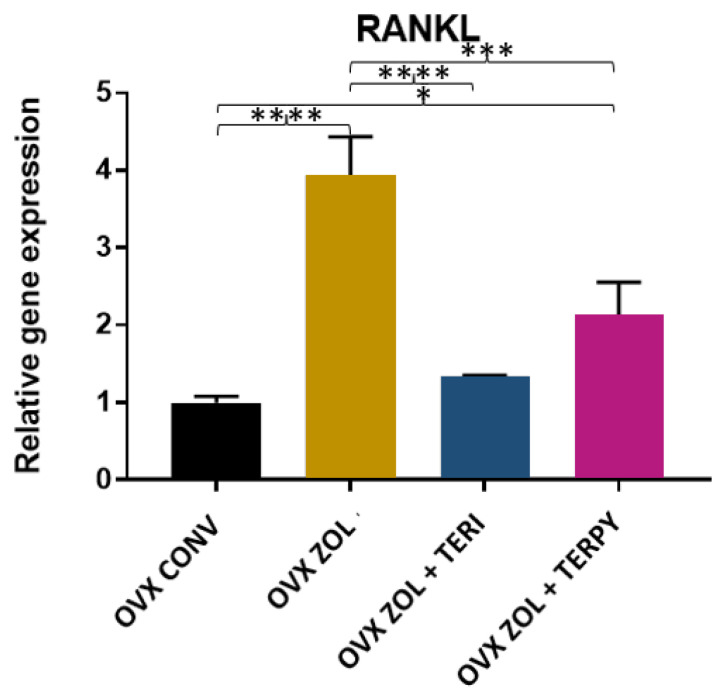
Relative gene expression of the RANKL gene for the OVX CONV, OVX ZOL, OVX ZOL + TERI, and OVX ZOL + TERPY groups. Mean values of OVX CONV (0.9967), OVX ZOL (3.933), OVX ZOL + TERI (1.333), and OVX ZOL + TERPY (2.133). *: statistically significant difference (*p* ≤ 0.05); ***: statistically significant difference (*p* ≤ 0.0005); and ****: statistically significant difference (*p* ≤ 0.00005).

There was a statistically significant difference in the gene expression of RANKL between the following groups: OVX CONV-OVX ZOL, OVX CONV-OVX ZOL + TERPY, OVX ZOL-OVX ZOL + TERI, and OVX ZOL-OVX ZOL + TERPY, with *p* < 0.005. However, there was no statistically significant difference with the OVX CONV-OVX ZOL + TERI and OVX ZOL + TERI-OVX ZOL + TERPY groups, as shown in Figure 10.

**Figure 11:**
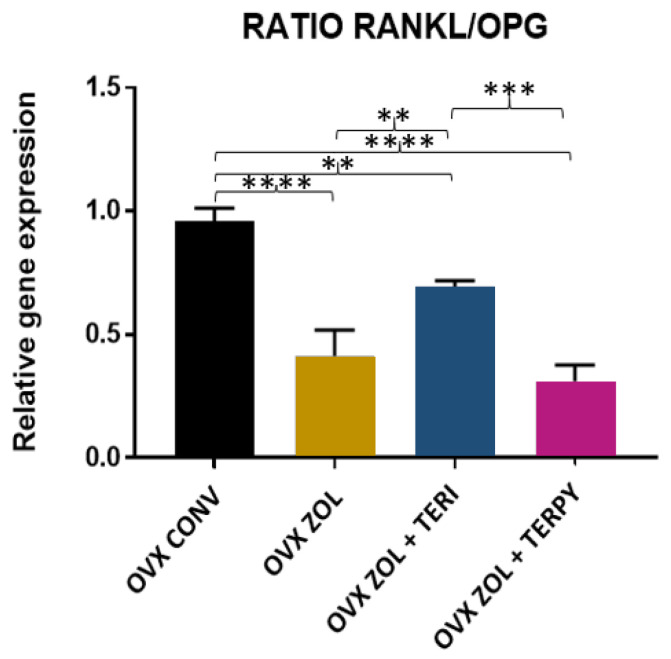
RANKL/OPG ratio for the OVX CONV, OVX ZOL, OVX ZOL + TERI, and OVX ZOL + TERPY groups. Mean values of OVX CONV (0.9567), OVX ZOL (0.4133), OVX ZOL + TERI (0.6933), and OVX ZOL + TERPY (0.3100). **: statistically significant difference (*p* ≤ 0.005); ***: statistically significant difference (*p* ≤ 0.0005); and ****: statistically significant difference (*p* ≤ 0.00005).

There was a statistically significant difference in the RANKL/OPG ratio between the following groups: OVX CONV-OVX ZOL, OVX CONV-OVX ZOL + TERI, OVX CONV-OVX ZOL + TERPY, OVX ZOL-OVX ZOL + TERI, and OVX ZOL + TERI-OVX ZOL + TERPY, with *p* < 0.005. However, there was no statistically significant difference with the OVX ZOL-OVX ZOL + TERPY group, as shown in Figure 11.

**Figure 12:**
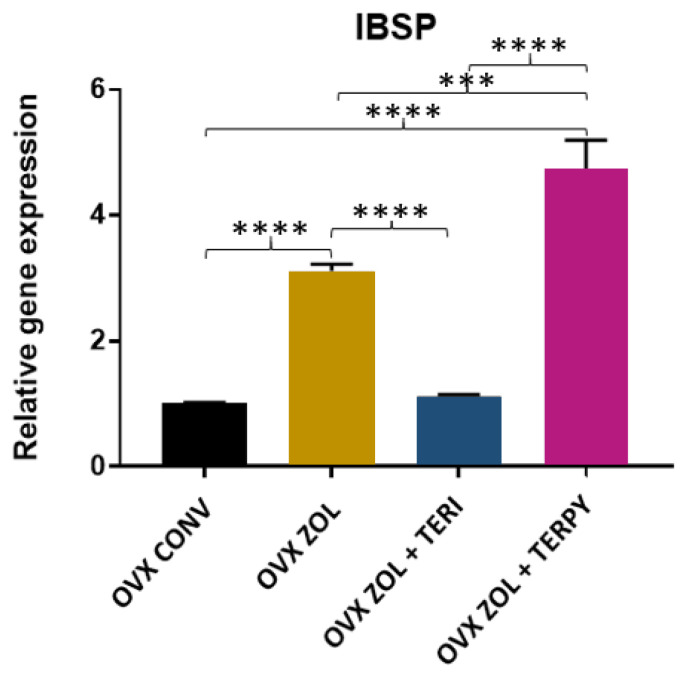
Relative gene expression of the IBSP gene for the OVX CONV, OVX ZOL, OVX ZOL + TERI, and OVX ZOL + TERPY groups. Mean values of OVX CONV (1.003), OVX ZOL (3.117), OVX ZOL + TERI (1.120), and OVX ZOL + TERPY (4.747). ***: statistically significant difference (*p* ≤ 0.0005); and ****: statistically significant difference (*p* ≤ 0.00005).

There was a statistically significant difference in the gene expression of IBSP between the following groups: OVX CONV-OVX ZOL, OVX CONV-OVX ZOL + TERPY, OVX ZOL-OVX ZOL + TERI, OVX ZOL-OVX ZOL + TERPY, and OVX ZOL + TERI-OVX ZOL + TERPY, with *p* < 0.005. However, there was no statistically significant difference with the OVX CONV-OVX ZOL + TERI group, as shown in Figure 12.

**Figure 13:**
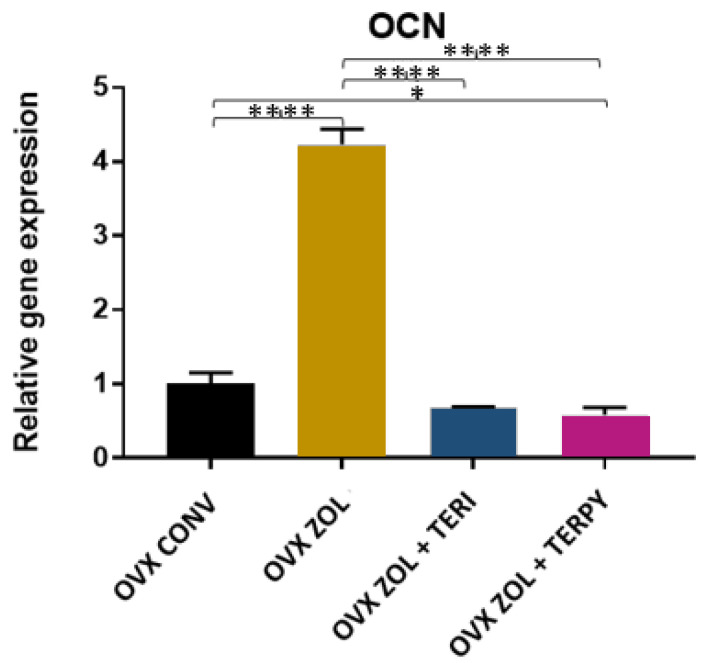
Relative gene expression of the OCN gene for the OVX CONV, OVX ZOL, OVX ZOL + TERI, and OVX ZOL + TERPY groups. Mean values of OVX CONV (1.007), OVX ZOL (4.233), OVX ZOL + TERI (0.6733), and OVX ZOL + TERPY (0.5867). *: statistically significant difference (*p* ≤ 0.05) and ****: statistically significant difference (*p* ≤ 0.00005).

There was a statistically significant difference in the gene expression of OCN between the following groups: OVX CONV-OVX ZOL, OVX CONV-OVX ZOL + TERPY, OVX ZOL-OVX ZOL + TERI, and OVX ZOL-OVX ZOL + TERPY, with *p* < 0.005. However, there was no statistically significant difference with the OVX CONV-OVX ZOL + TERI and OVX ZOL + TERI-OVX ZOL + TERPY groups, as shown in Figure 13.

**Figure 14:**
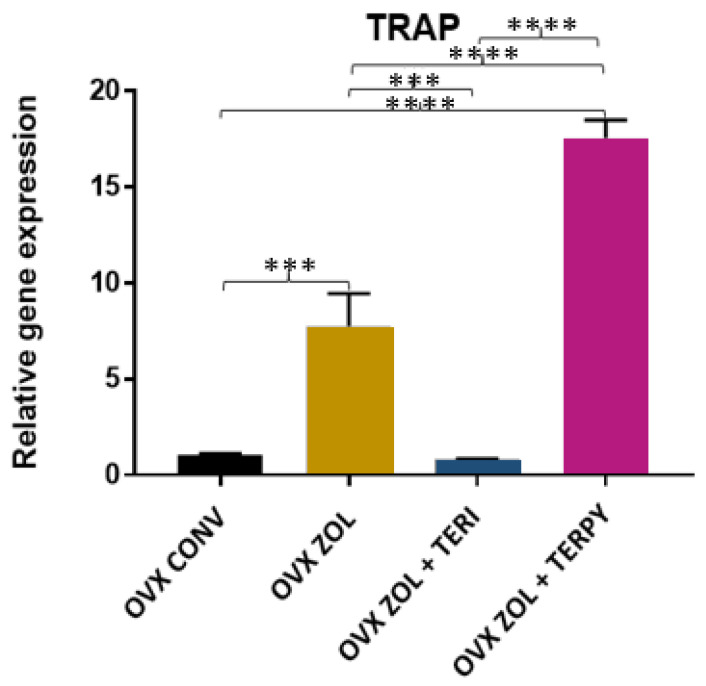
Relative gene expression of the TRAP gene for the OVX CONV, OVX ZOL, OVX ZOL + TERI, and OVX ZOL + TERPY groups. Mean values of OVX CONV (1.033), OVX ZOL (7.763), OVX ZOL + TERI (0.783), and OVX ZOL + TERPY (17.56). ***: statistically significant difference (*p* ≤ 0.0005); and ****: statistically significant difference (*p* ≤ 0.00005).

There was a statistically significant difference in the gene expression of TRAP between the following groups: OVX CONV-OVX ZOL, OVX CONV-OVX ZOL + TERPY, OVX ZOL-OVX ZOL + TERI, OVX ZOL-OVX ZOL + TERPY, and OVX ZOL + TERI-OVX ZOL + TERPY, with *p* < 0.005. However, there was no statistically significant difference with the OVX CONV-OVX ZOL + TERI group, as shown in Figure 14.

All the statistical analysis results for RT-PCR analysis can be found in the Supplementary Materials (Table S2).

## 3. Discussion

The indication of systemic bisphosphonates for osteoporotic patients has been under discussion in recent years with a greater focus on their true cost-effectiveness given their range of undesirable side effects on the body [25,26]. However, within the same scenario, the increasing life expectancy of the population worldwide has resulted in a higher prevalence of osteoporotic patients requiring oral rehabilitation with the installation of osseointegrable implants. Such patients, if left untreated, have bone tissue with cellular, structural, and biomechanical characteristics unfavorable for osseointegration [27]. Thus, the present study was aimed at attempting to locally improve the characteristics of bone tissue compromised by the presence of osteoporosis by surface functionalizing implants with combinations of bioactive molecules that promote complementary cellular responses. Some studies from our research group have already performed surface functionalization of implants using teriparatide and ruterpy, with good results [28,29]. However, the association of these biomolecules around implants aiming at their ordered release in models of osteoporotic rats, not only osteopenic ones, had not yet been studied.

In agreement with previously published results [17,18,19,20,21,22,23,30], the local peri-implant release of zoledronic acid in the OVX ZOL group resulted in a significant improvement in morphological parameters (bone tissue volume—BV/TV, number of bone trabeculae—Tb.N, separation between bone trabeculae—Tb.S, total bone tissue porosity—Po.Tot, and bone–implant contact interface—I.S), histological parameters (greater peri-implant bone neoformation when compared to the OVX CONV group), and molecular parameters (greater expression of genes related to bone tissue formation and mineralization—OPG, IBSP, and OCN—and, simultaneously, of genes related to bone remodeling and osteoclastic activity—RANKL and TRAP, demonstrating a higher turnover of bone tissue and balance between its formation and reabsorption, as evidenced by a significant improvement in the OPG/RANKL ratio compared to the control group). These results confirm the efficacy of this drug as an anti-resorptive agent in the presence of osteoporosis. It also produced better results than alendronate when the latter was used around implants in estrogen-deficient rats [31].

However, when comparing the OVX ZOL group with the OVX ZOL + TERI and OVX ZOL + TERPY groups, there were no significant differences in the microcomputed tomography analysis for any of the parameters analyzed; in the qualitative histological analysis, on the other hand, the groups with double functionalization seemed to have a greater pattern of maturity and organization of the peri-implant bone tissue when compared to the OVX ZOL group—especially the OVX ZOL + TERI group. This suggests that although the association between the drugs did not promote bone improvement in a structural scope, histologically, there was a positive response to such association. In the real-time PCR analysis, we also observed a significant increase in the expression of all the genes studied, except for osteocalcin (OCN), in the OVX ZOL + TERPY group compared to the OVX CONV group. This suggests that such surface, like the OVX ZOL surface, also promotes a greater balance in the bone turnover process in the peri-implant region in the presence of osteoporosis. This balance resulted in greater formation and organization of bone tissue, as evidenced by histological and three-dimensional microtomographic analyses. However, when observing the results of the OVX ZOL + TERI group in the real-time PCR analysis, we obtained a different pattern from that observed in the other surface functionalization groups: there was a slight numerical, nonsignificant increase in gene expression of all the studied genes compared to the OVX CONV group, and a significant decrease when compared to the OVX ZOL and OVX ZOL + TERPY groups. This pattern of results leads to the conclusion that the combination of zoledronic acid (an antiresorptive drug) with teriparatide (a bone anabolic drug) may have promoted an acceleration of the cellular and molecular events involved in the peri-implant repair process. While there were no peaks of gene expression of the studied genes at 28 postoperative days, there was the presence of organized and mature bone tissue microscopically and qualitatively abundant morphologically.

Finally, when comparing the OVX ZOL + TERI and OVX ZOL + TERPY groups, no statistically significant differences were observed in the microcomputed tomography analysis, but a slight increase in the values of the analyzed parameters was noted in the OVX ZOL + TERI group compared to the OVX ZOL + TERPY group. This increase was confirmed by the qualitative histologic analysis of the peri-implant tissue.

According to Kitagawa et al. [32], when five layers of functionalization are applied to implants using the layer-by-layer technique (very similar to the dip-coating technique used in this study), a single layer of 37 nm thickness is formed, allowing for 14 days of continuous release of the chosen drug into the bone tissue. Following the same reasoning, the formation of six functionalization layers (performed in the OVX ZOL + TERI and OVX ZOL + TERPY groups) would result in the formation of a slightly thicker single layer and, probably, a slightly longer drug release time, but similar to that reported in the study. The literature shows, in studies also using the model of placement of osseointegratable implants in osteoporotic rats, that mature bone tissue can be found after approximately 21 days of surgery [33], and the characteristics of this tissue are influenced by biochemical and cellular events that occurred before this period.

Indeed, the use of drug delivery systems has already been explored in the literature. However, their application is not limited to improving compromised bone tissue: recent work has used these devices to release various molecules at the periphery of bone tumors, to aid in their treatment. For example, while one study showed promising results in the repair of critical bone defects using 3D-printed polyetheretherketone scaffolds coated with bioactive hydroxyapatite and anticancer drugs [34], another showed a positive impact on osteosarcoma tumors when treated with methylmethacrylate and on Sarcoma-180 tumors when exposed to chitosan nanoparticles [35].

The presence of the biomolecules used in this study in the peri-implant bed during important periods for bone formation was demonstrated both by the microcomputed tomography analysis, which showed the superiority of the surface functionalization groups over the control, and by the descriptive histological analysis, which, as already explained, showed greater formation, maturation, and organization of the bone tissue in the groups with double functionalization compared to the group functionalized with zoledronic acid alone, and also, in this case, greater bone formation than in the control group.

With regard to the limitations of this study, it is worth mentioning the lack of subsequent euthanasia periods, which prevented the study of the consolidation of the positive responses obtained in the bone tissue with the long-term combination of zoledronic acid + teriparatide; the 95% statistical power; and the lack of a functional analysis of the implant, which would allow for the study of the bone responses when subjected to forces similar to the masticatory forces in the animals used in this research.

## 4. Materials and Methods

### 4.1. Animals

The procedures to be performed in this study were submitted for review to the Research Ethics Committee on the Use of Animals (CEUA) of this institution, and after its approval (protocol number 0520-2022), they were initiated. Forty-four adult female rats (Rattus norvegicus albinus, Wistar), aged 3 to 4 months at the beginning of the experiment, obtained from the central animal facility of the Araçatuba School of Dentistry—Júlio de Mesquita Filho São Paulo State University—UNESP, were used. Throughout the experiment, the rats were maintained in a controlled environment with a stable temperature (22 ± 2 °C) and a 12 h light/dark cycle, and provided with ad libitum access to solid food (balanced diet [Nuvilab, Curitiba, PR, Brazil]) and water. Ninety days after bilateral ovariectomy, the animals were randomly divided into 4 groups (OVX CONV, OVX ZOL, OVX ZOL + TERI, and OVX ZOL + TERPY) according to the surface of the implants to be implanted, as follows: OVX CONV = group in which conventional implants without surface functionalization were placed; OVX ZOL = group in which implants functionalized with zoledronic acid by the dip-coating technique were placed; OVX ZOL + TERI = group in which implants functionalized with zoledronic acid and teriparatide by the dip-coating technique were placed; and OVX ZOL + TERPY = group in which implants functionalized with zoledronic acid and ruterpy by the dip-coating technique were placed (Table 1), totaling n = 44 rats, with 11 rats per group.

### 4.2. Experimental Model Design

Female Wistar rats (Rattus norvegicus albinus, Wistar, Philadelphia, PA, USA) undergoing ovariectomy surgery were used to evaluate peri-implant bone repair. The locally administered drugs were zoledronic acid, zoledronic acid + teriparatide, and zoledronic acid + ruterpy. These substances were incorporated into the implants to be placed in the tibial metaphyses of the rats. The proposed analyses for this study were micro-computed tomography (Micro-CT) analysis and qualitative histologic analysis.

On day 0, the rats selected for the experiment underwent bilateral ovariectomy surgery. After 90 days (day 90), implant installation was performed in the tibial metaphyses. At 28 days after implant placement (day 118), the animals were euthanized, and one tibia from each animal was randomly reserved for micro-computed tomography scanning and the other for histological analysis. For this purpose, the animals were identified by a numerical sequence and randomly assigned to 1 of 2 groups (right tibia—M-CT/left tibia—H and right tibia—H/left tibia—M-CT), with randomization performed using Excel 2016 software.

### 4.3. Implants Used in This Research

Commercially pure-grade IV titanium implants based on the concept of double-acid etching (Medens^®^, Itu, SP, Brazil) with a diameter of 1.4 mm and height of 2.7 mm were used. The implants were sterilized by gamma irradiation.

### 4.4. Drug Treatment on the Implant Surface

The implants were functionalized using the dip-coating technique, in which they are immersed in a solution containing the desired molecule to form layers around them. For this purpose, their connectors (internal hexagon) were coupled with pipette tips of the appropriate size (for the study in question, 100 mL tips were used), which were then stabilized by the base in an alginate-based device. This setup ensured that the implants were not touched during or after the functionalization process, thus guaranteeing the formation of uniform layers.

Thus, 5 or 6 layers were applied around the implants (depending on the experimental group), as needed based on preliminary tests (a greater number of layers ensured that the surface functionalization was not lost during implant insertion into the surgical site). The molecules used for functionalization were zoledronic acid, ruterpy, and teriparatide, depending on the group. For implants to be placed in the OVX ZOL group, 5 layers of zoledronic acid functionalization were performed (5 dips with a 1-day interval between them), while for the OVX ZOL + TERI and OVX ZOL + TERPY groups, 6 layers of functionalization were performed (6 dips also with a 1-day interval between them), with the first 3 layers being of teriparatide or ruterpy, depending on the group, and the last 3 layers being zoledronic acid. After drying all the desired layers, the implants were placed in the animals of their respective groups.

The concentrations of the solutions containing teriparatide (PTH 1-34) and ruterpy were already established. The teriparatide solution consisted of 20 μL of teriparatide diluted in 50 mL of Milli-Q^®^ water + 50 mL of DMSO (dimethyl sulfoxide), while the ruterpy solution consisted of 10 mL of a more concentrated ruterpy solution diluted in 40 mL of Milli-Q^®^ water + 50 mL of DMSO. The more concentrated solution consisted of 25 mg of ruterpy diluted in 50 mL of Milli-Q^®^ water. The concentration of the zoledronic acid solution was determined based on other studies in the literature [20,21,22,23], and consisted of 1 mg of zoledronic acid diluted in 1 mL of Milli-Q^®^ water + 99 mL of DMSO. Once prepared, the solutions were stored in 100 mL beakers, kept at a temperature of 18 °C, and discarded at the end of the functionalization process.

### 4.5. Experiments with the Osteoporosis Experimental Model

After the functionalization of the implants, in vivo experiments were performed in the experimental osteoporosis model proposed for this study. The aim was to evaluate the synergistic effect between locally administered drugs and to characterize, in a general way, the initial responses of the peri-implant bone tissue in terms of bone formation, resorption, and mineralization events.

It is worth noting that the model of implant placement in the tibial metaphysis of the rats was used to evaluate the effect of osteoporosis on these bones and the possible improvement of this condition through surface functionalization of the implants with zoledronic acid, zoledronic acid + teriparatide, and zoledronic acid + ruterpy by the dip-coating technique.

### 4.6. Estrous Cycle

To ensure that the rats used in the experiments were cycling normally, they were individually caged, and 1–2 drops of saline solution were introduced into the vagina daily. The solution was then aspirated and placed on a histological slide for immediate microscopic reading (Long and Evans technique, 1922) [36], aiming to identify the phases of the estrous cycle. The rats were used after obtaining 2 to 3 regular estrous cycles. This technique was used again after bilateral ovariectomy surgery to validate the efficacy of the procedure.

### 4.7. Ovariectomy

After an eight-hour fast, the rats were anesthetized intramuscularly with 5 mg/kg of xylazine hydrochloride (2% xylazine hydrochloride injectable, Syntec, Tamboré-SP, Brazil) and 50 mg/kg of ketamine hydrochloride (ketamine hydrochloride injectable, Vetnil, Louveira-SP, Brazil) and immobilized on a surgical board. After trichotomy of the surgical area, the area to be incised was aseptically prepared with Povidone-Iodine Germicidal Solution (10% Povidone-Iodine, Riodeine Germicidal, Rioquímica, São José do Rio Preto-SP, Brazil), combined with topical Povidone-Iodine. In the lateral decubitus position, an approximately 1 cm incision was made on the flanks, followed by dissection through the subcutaneous tissue planes and then through the peritoneum to access the abdominal cavity. The ovaries and uterine horns were then located, ligated with monofilament suture (Nylon 5-0, Ethicon, Johnson, São José dos Campos-SP, Brazil), and surgically removed. Suturing was performed in layers, with interrupted sutures using monofilament suture (Nylon 5-0, Ethicon, Johnson, São José dos Campos, SP, Brazil) for inner levels and multifilament sutures (Silk 4-0, Ethicon, Johnson, São José dos Campos, SP, Brazil) for the outer- level. As a prophylactic measure, 0.2 mL of Benzathine Penicillin G (Pentabiótico Veterinary Small Size, Fort Dodge Animal Health Ltd.a., Campinas, SP, Brazil) was administered intramuscularly to all animals immediately after surgery [37].

### 4.8. Surgery to Place Osseointegratable Implants in the Tibia

Ninety days after bilateral ovariectomy or sham surgery (day 90), the rats underwent implant placement in their tibial metaphysis. Following the same protocol used for bilateral ovariectomy surgery, the animals were fasted for 8 h prior to surgery and anesthetized intramuscularly with 50 mg/kg of ketamine hydrochloride (ketamine hydrochloride injectable, Vetnil, Louveira, SP, Brazil) and 5 mg/kg of xylazine hydrochloride (2% xylazine hydrochloride injectable, Syntec, Tamboré, SP, Brazil). After anesthesia, trichotomy was performed on the medial part of both right and left tibias. The incision area was aseptically prepared with Povidone-Iodine Germicidal Solution (10% Povidone-Iodine, Riodeine Germicidal, Rioquímica, São José do Rio Preto, SP, Brazil) combined with topical Povidone-Iodine.

**Figure 15:**
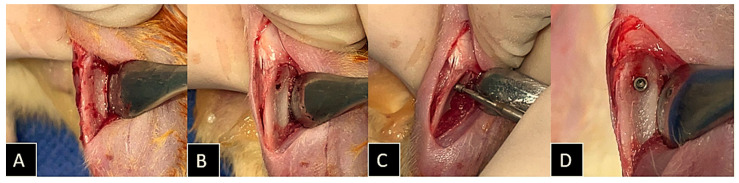
Procedure for implant installation in the tibial metaphyses of the animals. (**A**) Access to the tibial metaphysis; (**B**) bicortical milling of the bone bed with a 1.1 mm diameter bur; (**C**) implant placement using a digital wrench; and (**D**) implant placed in the correct position.

Using a no. 15 blade (Feather Industries Ltd.a, Tokyo-To, Japan), an approximately 1 cm incision was made in the region of the left and right tibial metaphyses, and then the soft tissues were dissected and retracted with periosteal elevators, exposing the bone to receive the implants. A total of 88 commercial-grade IV titanium implants, 1.4 mm in diameter and 2.7 mm in height, sterilized by gamma irradiation, were placed (2 per animal). Milling was performed in both cortices with a 1.1 mm diameter spiral drill attached to an electric motor (BLM600^®^; Driller, São Paulo, SP, Brazil) at a speed of 1000 rpm, under irrigation with isotonic sodium chloride solution 0.9% (Physiological^®^, Laboratórios Biosintética Ltd.a^®^, Ribeirão Preto, SP, Brazil), and a 20:1 reduction contra-angle handpiece (Angular piece 3624N 1:4, Head 67RIC 1:4, KaVo^®^, Kaltenbach & Voigt GmbH & Co., Biberach, Germany) (Figure 15).

The tissues were sutured in layers with interrupted sutures, using monofilament sutures (Nylon 5.0, Ethicon, Johnson, São José dos Campos, SP, Brazil) for the inner layers and multifilament sutures (Silk 4-0, Ethicon, Johnson, São José dos Campos, SP, Brazil) for the outermost layer. Immediately postoperatively, each animal received an intramuscular dose of 0.2 mL of Benzathine Penicillin G (Pentabiótico Veterinary Small Size, Fort Dodge Animal Health Ltd.a., Campinas, SP, Brazil).

### 4.9. Euthanasia

The animals were euthanized by anesthetic overdose 28 days after implant placement (day 118). Bilateral tibiae were harvested for micro-computed tomography (Micro-CT) and histologic analyses.

### 4.10. Processing of Calcified Tissues

After processing the samples for calcified tissues (fixation in 10% buffered formalin for 48 h—Reagentes Analíticos^®^, Dinâmica Odonto-Hospitalar Ltd.a, Catanduva, SP, Brazil—washing in running water for 24 h, and storage in 70% alcohol), micro-computed tomography (Micro-CT) scanning and analysis were performed. This analysis aimed to morphologically and quantitatively evaluate the process of peri-implant bone repair and formation and compare the parameter values obtained among the different experimental groups. The evaluated parameters followed the guidelines for the evaluation of bone microarchitecture in rodents using micro-computed tomography, published by Bouxsein et al. [38].

### 4.11. Three-Dimensional Microtomographic Evaluations (Micro-CT)

The specimens were scanned with the SkyScan microtomograph (SkyScan 1272 Bruker MicroCT, Aatselaar, Belgium, 2003), using 8 μm thick slices (90 kV and 111 μA), with an Al 0.5 mm + Cu 0.038 filter and a rotation step of 0.4 mm, pixel size of 2016 × 1344 μm, and acquisition time of 1 h and 56 min. The X-ray projection images obtained from the specimens were stored and reconstructed to determine the region of interest using NRecon software (SkyScan, 2011; Version 1.6.6.0), with a smoothing of 5, artifact ring correction of 10, beam hardening correction of 40%, and the image conversion range varied from 0.0 to 0.14. In the Data Viewer software (SkyScan, Version 1.4.4 64-bit), the images were reconstructed and evaluated in the transaxial plane.

For morphometric analysis in 3D, the CTAnalyser software—CTAn (2003-11SkyScan, 2012 Bruker MicroCT Version 1.12.4.0) was used. Standardization of the region of interest (ROI) was performed using the BIC method, which includes the peri-implant bone region in contact with the implant. Using the BIC method, 150 slices corresponding to the medullary portion were demarcated, starting from the disappearance of the upper cortical bone and appearance of medullary bone. The CTAn software analyzed and measured the image according to a grayscale threshold. The threshold used in the analysis was 20–120 gray levels, which allowed the calculation of the bone volume and porosity around the implants. Based on this conversion, the software performed morphometric calculations applied to the ROI using parameters such as the (BV/TV) percentage of bone volume; (Tb.N) number of trabeculae; (Tb.Sp) trabecular separation; (Tb.Th) trabecular thickness; (Po.Tot) total bone tissue porosity; and (I.S) bone-to-implant interface.

For educational and illustrative purposes, reconstruction and manipulation of the samples in three dimensions were also performed using CTvox software (SkyScan, Version 2.7).

### 4.12. Molecular Analysis (Real-Time PCR)

Immediately after removal of the implants from the tibiae using a reverse torque, the peri-implant bone was harvested with mini gouge forceps, maintaining at least 0.5 cm on each side of the peri-implant space and preserving the bone in contact with the implant threads. The bone was carefully washed in phosphate-buffered saline, frozen in liquid nitrogen, and stored at −80 °C to extract the total RNA using a Trizol reagent (Life Technologies: Invitrogen, Carlsbad, CA, USA). After RNA integrity, purity, and concentration analysis, cDNA was created using 1 µg RNA via a reverse transcriptase reaction (M-MLV reverse transcriptase: Promega Corporation, Madison, WI, USA). Sample cDNA was pipetted onto the array PCR plate with TaqMan Fast Advanced Mastermix (Applied Biosystems, Foster City, CA, USA) to detect genes involved in the bone repair process (TaqMan Array Fast 96 well plate, Applied Biosystems). Real-time PCR was performed on a Step One Plus real-time PCR detection system (Applied Biosystems) under the following conditions: 50 °C (2 min), 95 °C (10 min), 40 cycles of 95 °C (15 s), and 60 °C (1 min), followed by a standard denaturation curve. The relative gene expression was calculated by referencing the mitochondrial ribosomal protein expression and normalized by the gene expression of tibia fragments undergoing repair during different experimental periods (∆∆CT method). The assay was performed in quadruplicate, and the genes evaluated were osteoprotegerin (OPG—Tnfrsf11b—Rn00563499_m1), receptor activator of nuclear factor kappa B ligand (RANKL—Tnfrsf11—Rn00589289_m1), integrin binding sialoprotein (IBSP—Rn00561414_m1), osteocalcin (OCN—Bglap—Rn00566386_g1), and tartarate-resistant acid phosphatase (TRAP—Rn01424636_m1), with beta-actin (β2-Microglobulin, BACT—Actb—Rn00667869_m1) as constitutive, as shown in Table 2. The values obtained from the proposed analyses were submitted to statistical analysis using GraphPad Prism 7.03 software. The test for homoscedasticity was the Shapiro–Wilk test, and the normal distribution of the data was confirmed for all parameters analyzed. Next, the one-way ANOVA test was performed. In the sequence, the Tukey post-test was performed with the significance level *p* < 0.05, to indicate the differences between the groups.

### 4.13. Processing of Decalcified Tissues

After processing the samples for decalcification (fixation in 10% buffered formalin for 48 h—Reagentes Analíticos^®^, Dinâmica Odonto-Hospitalar Ltd.a, Catanduva, SP, Brazil, washing in running water for 24 h and successive changes of 10% EDTA until decalcification—approximately 16 changes or 8 weeks), the specimens were washed again in running water for 24 h, dehydrated in xylene, embedded in paraffin, sectioned to obtain 6μm thick sections, and mounted on slides. They were then stained with hematoxylin and eosin for qualitative histologic analysis.

### 4.14. Qualitative Histological Analysis

After decalcification processing with 10% EDTA, the tibias from each group were washed again in running water for 24 h, dehydrated in an ascending series of alcohols, cleared in xylene, and embedded in paraffin. From the obtained blocks, 6 μm sections were prepared using a microtome (Leica RM2125 RTS, Leica BioSystems, Nussloch, Germany). Subsequently, the sections were mounted on histological slides and stained with hematoxylin for 8 min and eosin 1% for 30 s. After staining, coverslips were mounted on slides, and they were observed under an optical microscope (Leica DM 750, Leica BioSystems, Nussloch, Germany) at 4×, 20×, and 40× magnifications to capture images of the central region of the peri-implant area using a digital camera for microscopic image capture (Leica MC 170 H, Leica BioSystems, Nussloch, Germany). (D). The images were saved in JPG format.

### 4.15. Statistical Analysis

The data obtained from the analysis of microtomography (Micro-CT) and real-time PCR were subjected to statistical analysis using GraphPad Prism 7.03 software. After applying the Shapiro–Wilk test for homoscedasticity and confirming the normal distribution of the data, they were subjected to the parametric test of one-way analysis of variance (one-way ANOVA), followed by Tukey’s post-test, when necessary, to determine the differences observed between the evaluated groups. The significance level was set at 5% (*p* < 0.05).

The dependent variable used to determine the sample size was BV/TV, and the data were taken from the article by Gomes-Ferreira et al. [39]. The sample size for this study for each group was determined using the power test via the website http://www.openepi.com/SampleSize/SSMean.htm (OpenEpi, Version 3, open-source calculator, accessed on 9 November 2022), and based on previously published results, the means used for the calculation were 33.37 and 43.54, with standard deviations of 8.53 and 5.77, at a significance level of 5% and a power of 95% in a one-tailed hypothesis test. As such, a sample size of 11 animals per group was required. The estimate for each outcome measure was made to achieve a power of 0.8 and an alpha error of 0.05.

## 5. Conclusions

All surface functionalization groups outperformed the control group, with better results for the OVX ZOL + TERI group. However, further analysis is needed to confirm this finding and investigate the long-term consolidation of bone formation.

## 6. Patents

BR 10 2021 025891 8 process—“Funcionalização da superfície de implantes com molécula doadora de óxido nítrico através da técnica layer by layer como estratégia para a melhora do processo de reparo periimplantar em ratas”(870210118781 petition).

BR 10 2021 019548 7 process—“Funcionalização da superfície de implantes com anabólico ósseo através da técnica de layer by layer como estratégia para a melhora do processo de reparo periimplantar em ratas” (870210089896 petition).

## Figures and Tables

**Table 1:** Experimental groups according to the surface of the implants to be placed.

Implant Surface	Number of Animals	Description of the Group
OVX CONV	11	Group in which conventional implants without surface functionalization were placed.
OVX ZOL	11	Group in which implants functionalized with zoledronic acid by the dip-coating technique were placed.
OVX ZOL + TERI	11	Group in which implants functionalized with zoledronic acid and teriparatide by the dip-coating technique were placed.
OVX ZOL + TERPY	11	Group in which implants functionalized with zoledronic acid and ruterpy by the dip-coating technique were placed.

**Table 2:** TaqMan probes for real-time PCR.

Gene	Gene Name	Identification
*OPG*	*Tnfrsf11b*	Rn00563499_m1
*RANKL*	*Tnfrsf11*	Rn00589289_m1
*IBSP*	*IBSP*	Rn00561414_m1
*TRAP*	*TRAP*	Rn01424636_m1
*OCN*	*Bglap*	Rn00566386_g1
*BACT*	*Actb*	Rn00667869_m1

## Data Availability

Data are contained within this article.

## Supplementary Materials

The following supporting information can be downloaded at: https://www.mdpi.com/article/10.3390/ijms25168963/s1.
